# CAR-T cell therapy: Where are we now, and where are we heading?

**DOI:** 10.1097/BS9.0000000000000173

**Published:** 2023-11-02

**Authors:** Jia-Yi Wang, Liang Wang

**Affiliations:** a Department of Hematology, Beijing Tongren Hospital, Capital Medical University, Beijing 100730, China

**Keywords:** Chimeric antigen receptor T-cell therapy, Cytokine release syndrome, Efficacy, Hematology

## Abstract

Chimeric antigen receptor (CAR)-T-cell therapies have exhibited remarkable efficacy in the treatment of hematologic malignancies, with 9 CAR-T-cell products currently available. Furthermore, CAR-T cells have shown promising potential for expanding their therapeutic applications to diverse areas, including solid tumors, myocardial fibrosis, and autoimmune and infectious diseases. Despite these advancements, significant challenges pertaining to treatment-related toxic reactions and relapses persist. Consequently, current research efforts are focused on addressing these issues to enhance the safety and efficacy of CAR-T cells and reduce the relapse rate. This article provides a comprehensive overview of the present state of CAR-T-cell therapies, including their achievements, existing challenges, and potential future developments.

## 1. INTRODUCTION

Chimeric antigen receptor (CAR)-T-cell therapy is an adoptive cell therapy that bypasses major histocompatibility complex-dependent immune tolerance and tumor escape; it targets a broad array of antigens (proteins, carbohydrates, and glycolipids),^1^ and has higher durability than conventional chemotherapy, presenting a promising form of immunotherapy. It targets cancer cells through genetically modified T cells from patients or healthy donors, meeting the current trends of precision and personalized medicine. CAR-T cells have shown promising results in the treatment of B-cell malignant diseases, and 9 CAR-T-cell products targeting B-cell malignant tumors or myeloma are available.

CAR-T cells have toxic side effects that cannot be ignored, with the cytokine release syndrome (CRS) and immune effector cell–associated neurotoxicity syndrome (ICANS) being the most common. Management of CAR-T-cell toxicity is an important aspect of CAR-T-cell therapy. Current CAR-T-cell developments continue to reduce toxicity and improve efficacy.

## 2. WHERE ARE WE NOW?

### 2.1 Structure and function of CAR-T cells

**Figure 1.**
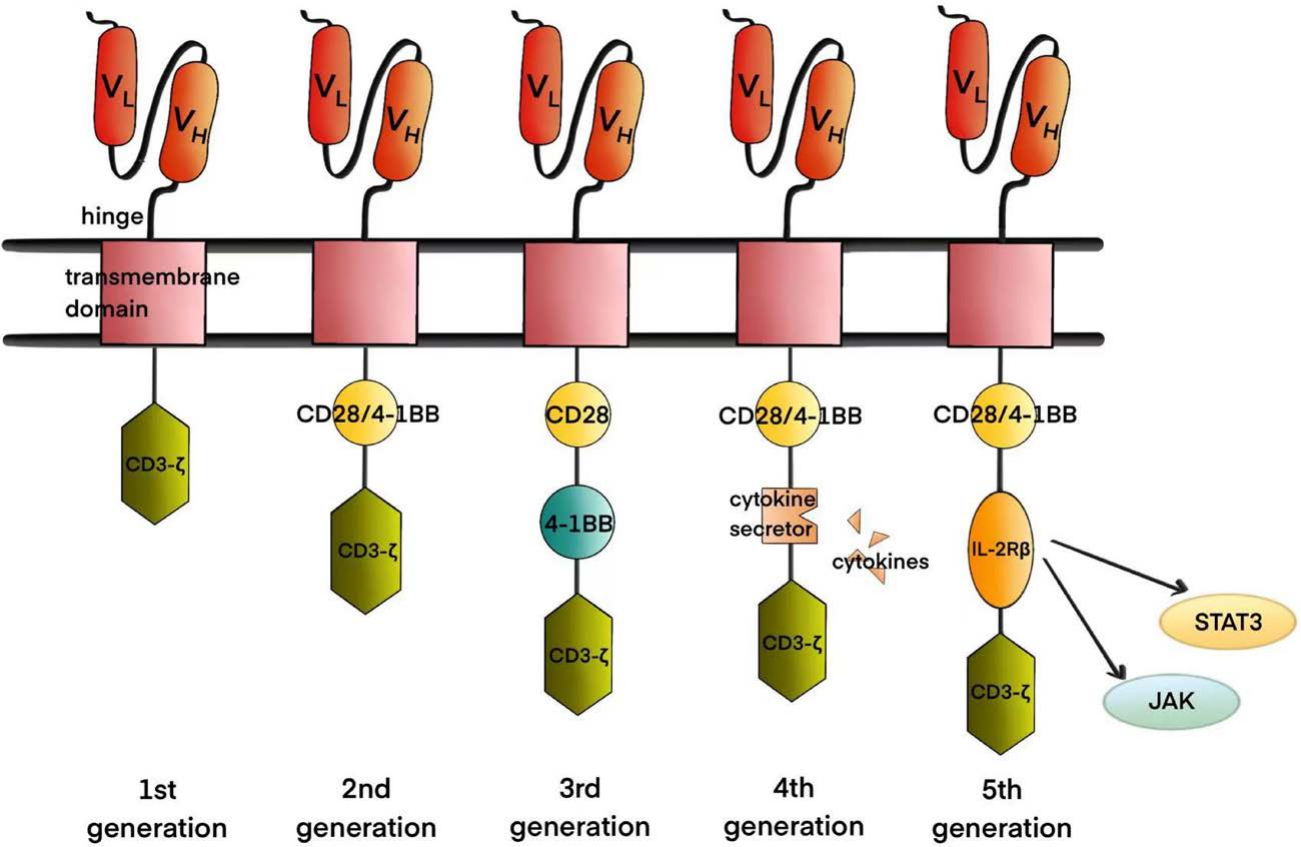
Five generations of CAR. The first-generation CAR-T-cell structure consists of only 1 antigen recognition domain, scFv, a hinge region, a transmembrane domain, and an intracellular activation domain, whereas the second-generation CAR-T cells have an additional co-stimulatory domain, and the third-generation CAR-T cells have 2 different co-stimulatory domains. Fourth-generation CAR-T cells secrete cytokines via modifications. Fifth-generation CAR-T cells are the latest generation of CAR-T cells, with greater safety and controllability. CAR = chimeric antigen receptor, IL = Interleukin.

CAR is fundamentally composed of distinct segments: the extracellular, hinge, transmembrane, and intracellular structural domains.^2^ Specifically, the extracellular structural domain, referred to as the antigen-binding domain, originates from the variable heavy chain (VH) and variable light chain (VL) of monoclonal antibodies. These chains are interconnected by flexible junctions, forming a single-chain variable fragment (scFv), which primarily serves to facilitate the recognition of pertinent antigens on the surface of tumor cells.^3^ The mode of interaction between the VH and VL chains and the relative positions of the regions that determine complementarity can influence the affinity and specificity of CARs for target antigens.^4^ The hinge or spacer region is a part of the extracellular structure that extends from the transmembrane domain to the binding unit.^5^ Its role is related to the function of CAR-T cells, in addition to linking the extracellular and transmembrane structural domains. Okuno et al found that hinge region length modifications helped distinguish normal from tumor cells.^6^ Additionally, reducing the flexibility of the hinge region attenuated the over-activation of CAR-T cells and improved survival under a high-tumor load.^7^ The transmembrane structural domain acts as a signaling channel for the intracellular spacer and is usually derived from CD3-ζ, CD4, CD8, or CD28 molecules.^2^ Its main function is to anchor CAR molecules to the T-cell membrane, and studies have shown that it regulates CAR signaling by controlling the level and stability of CAR expression, which plays a role in signaling or synapse formation and is associated with the dimerization of endogenous signaling molecules.^8–11^ The intracellular structural domain is primarily composed of co-stimulatory domains and signal transduction regions. In CAR engineering, the implications of CAR co-stimulation are a crucial focus, with the ultimate objective of fabricating CAR architectures optimizing intracellular structural domains for functionality (Fig. 1).^5^

### 2.2. Development of CAR-T cells

Different generations of CAR-T cells have the same basic structure. They differ mainly in their intracellular structural domains and the cytokines and ligands introduced. First-generation CAR-T cells have the simplest structure, containing only an antigen recognition domain, scFv, hinge region, transmembrane domain, and intracellular CD3-ζ activation domain.^12^ The absence of a co-stimulatory domain causes a notably short duration of survival in clinical trials, constraining the cytotoxic potential. The integration of a co-stimulatory domain such as CD28 or 4-1BB into the initial generation of CAR-T cells constitutes the advent of second-generation CAR-T cells, enhancing proliferative capacity and cytokine secretion.^13^ Presently, all commercially available CAR-T-cell products are second-generation. Third-generation CAR-T-cell designs encompass a dual co-stimulatory framework that incorporates distinct molecules, namely CD28 and 4-1BB. Theoretically, this configuration has the potential for heightened cytotoxicity and prolonged therapeutic effectiveness compared with second-generation equivalents. However, current evidence does not substantiate their superior clinical performance compared with their second-generation counterparts.^14,15^ Fourth-generation CAR-T cells, commonly referred to as TRUCK or armored CAR-T cells, are engineered to enhance therapeutic potential through cytokine secretion, such as Interleukin(IL)-12, or the expression of anti–PD-1 antibodies or suicide genes. This augmentation improves the efficacy of CAR-T cells by circumventing inhibitory factors.^16,17^ In contrast to their second-generation counterparts, fourth-generation CAR-T cells exhibit enhanced expansion kinetics, heightened potency, and increased resistance to suppressive influences.^2^ Beyond the fourth-generation, fifth-generation CAR-T cells build on the second-generation CARs by including an IL-2 receptor β (IL-2Rβ) chain between CD3-ζ and CD28 or 4-1BB, providing a STAT3 binding site, which activates JAK-STAT signaling. Universal CAR-T (UCAR-T) cells, obtained from healthy donors, are genetically edited to disrupt T-cell receptor (TCR) and human leukocyte antigen class I genes with biotin-binding immune receptors or split, universal, programmable CARs, enabling recognition of a wide range of antigens.^18,19^ Emerging technologies, such as logic gating, are increasingly being applied to the design of CAR-T cells to overcome the shortcomings of conventional CAR-T (C-CAR-T) cells and enhance their safety and efficacy.

### 2.3. Manufacture of CAR-T cells

CAR-T-cell generation involves a sequential cascade of processes. In the initial step, T-cells are obtained from the peripheral blood of either the patient or donor. As in the peripheral blood stem-cell collection in hematopoietic stem-cell transplantation (HSCT), autologous CAR-T-cell acquisition presents unique hurdles due to the concurrent presence of cytopenia and compromised T-cell functionality in patients. This hinders and poses significant challenges to the efficient retrieval of T cells.^2,20^ Isolated monoclones undergo genetic modification to express CARs, followed by amplification. Subsequently, the modified products are cryopreserved and transported to a hospital for administration. Patients usually undergo lymphodepleting chemotherapy, also referred to as conditioning chemotherapy, which augments a favorable cytokine milieu while diminishing immunosuppressive cells.^21,22^ The effect of conditioning chemotherapy on lymphocyte counts correlates with and is necessary for the optimal efficacy of CAR-T cells.^23,24^ CAR-T cells are then reinfused, specifically recognizing the target antigen and rapidly proliferating to exert anti-tumor effects in vivo.^25^ Continuous monitoring of patients’ vital signs is essential throughout the reinfusion, as well as during the subsequent 2- to 3-week period, to promptly detect and intervene in any potential toxic adverse effects, including CRS and ICANS. Furthermore, a comprehensive long-term follow-up should be conducted to evaluate the efficacy of CAR-T treatment.

### 2.4 Clinical application of CAR-T-cell therapy

Currently, CAR-T-cell therapies are primarily approved for hematologic tumors in both domestic and international markets. Among these, 6 products utilize anti-CD19 CAR-T cells, targeting relapsed or refractory (R/R) B-cell lymphoma and B-cell acute lymphoblastic leukemia (B-ALL), whereas the remaining three products employ anti–B-cell maturation antigen (BCMA) CAR-T cells to treat R/R multiple myeloma (MM) (Table 1).

**Table 1:** Nine CAR-T cell products.

CAR-T production	Target	Application
Kymriah (tisagenlecleucel, tisa-cel)	CD19	R/R B-ALL, R/R DLBCL
Yescarta (axicabtagene ciloleucel, axi-cel)	CD19	R/R DLBCL
Tecartus (brexucabtagene autoleucel)	CD19	R/R MCL, R/R B-ALL
Abecma (idecabtagene vicleucel, ide-cel)	BCMA	R/R MM
Breyanzi (lisocabtagene maraleucel, liso-cel)	CD19	R/R DLBCL
Axicabtagene ciloleucel	CD19	R/R DLBCL
Relmacabtagene autoleucel	CD19	R/R DLBCL;
Carvykti (ciltacabtagene autoleucel, cilta-cel)	BCMA	R/R MM
CT103A, IBI326	BCMA	R/R MM

CAR = chimeric antigen receptor, R/R B-ALL = relapsed/refractory B-cell acute lymphoblastic leukemia, R/R DLBCL = relapsed/refractory diffuse large B-cell lymphoma, R/R MCL = relapsed/refractory mantle cell lymphoma, R/R MM = relapsed/refractory multiple myeloma.

#### 2.4.1 Diffuse large B-cell lymphoma

Diffuse large B-cell lymphoma (DLBCL), the most common aggressive non-Hodgkin lymphoma (NHL), has a first-line treatment regimen of R-CHOP (rituximab combined with immunochemotherapy with cyclophosphamide, adriamycin, vincristine, and prednisone), achieving 70% to 80% remission rates. However, 30% to 40% of patients are unresponsive or relapse, leading to a poor prognosis with a 2-year overall survival (OS) of only 20% to 40%.^26^ The development and application of CAR-T-cell therapy offer new hope for the treatment of R/R DLBCL.

Anti-CD19 CAR-T-cell therapies are the gold standard third-line therapy and are rapidly evolving as second-line therapy for the treatment of refractory or early relapses after R-CHOP. Currently, 3 CAR-T products are marketed for DLBCL treatment: axicabtagene ciloleucel (axi-cel), tisagenlecleucel (tisa-cel), and lisocabtagene maraleucel (liso-cel).^27^ The Food and Drug Administration (FDA) approved axi-cel and tisa-cel in 2017 and 2018, respectively, for patients with high-grade B-cell lymphoma or DLBCL which were relapsed or previously treated with 2 or more lines of some sort of chemotherapy. Liso-cel was approved in 2021. A series of clinical trials and real-world evidence have demonstrated the effectiveness of CAR-T-cell therapy in the treatment of R/R DLBCL. The ZUMA-1 trial showed that axi-cel has an objective remission rate (ORR) of 83%, a complete remission (CR) rate of 53%, and a median OS of more than 2 years in patients with heavily pretreated large B-cell lymphoma (LBCL).^28^ The results of the 5-year follow-up showed a median OS of 25.8 months and a 5-year OS rate of 42.6%.^29^ The ZUMA-7 trial used axi-cel versus standard of care (SOC) as second-line therapy for patients with R/R LBCL, and showed a median event-free survival (EFS) of 8.3 months versus 2.0 months, an ORR of 83% versus 50%, and a CR rate of 65% versus 32%, in the axi-cel and SOC groups, respectively.^30^ ZUMA-12 was the first study to evaluate axi-cel as a first-line regimen for high-risk LBCL, with an ORR of 89% and a CR rate of 78%.^31^ A study from the US Lymphoma CAR-T Consortium reported an ORR of 82% and a CR rate of 64% for axi-cel in a SOC setting for approved indications.^32^ In the United States, a study of axi-cel for the treatment of LBCL reported an ORR of 73% and a CR rate of 56%. The median OS and progression-free survival (PFS) were 21.8 and 8.6 months, respectively.^33^

#### 2.4.2 Acute lymphoblastic leukemia

ALL shows high cure rates in children, with a 5-year OS rate of over 90%, but the long-term survival rate in adults is only 35% to 45%, with most relapses due to chemotherapy resistance.^34,35^ The prognosis for R/R ALL subtypes is much worse than for primary ALL, with a long-term OS rate of 15% to 50%.^36^ In recent years, CAR-T-cell therapy has made good progress in the treatment of R/R ALL, with the most studied target being CD19. In August 2017, the FDA approved the first CD19-CAR-T-cell product, tisa-cel, for the treatment of ALL in children and adolescents. In a single-center phase I-IIa study of tisa-cel, the overall remission rate was 81% at 3 months, and the event-free and OS rates at 6 months were 73% and 90%, respectively.^37^

Like CD19, CD22 is also a well-studied target in ALL. Of the adults and children with B-ALL, 90% express CD22. A phase I study of CD22^+^ CAR-T cells showed a dose-dependent CR rate, with 73% of the patients receiving higher doses achieving CR.^38^ A larger study demonstrated the safety and feasibility of using anti-CD22 CAR-T-cells. The incidence of CRS was 86.2%, with 90% grade 1-2 CRS, mild transient neurotoxic symptoms, and a CR rate of 70%. The median OS rate was 13.4 months.^39^ The management of associated toxic reactions and relapse after CAR-T-cell treatment of R/R B-ALL remains a challenge. In addition, greater follow-up is required to obtain more clinical data to confirm its long-term efficacy.

#### 2.4.3 Multiple myeloma

MM is largely incurable, and almost all patients eventually relapse, with remission times shortened with treatment options.^40^ CAR-T-cell therapy is a promising new treatment option for patients with R/R myeloma. The most widely studied MM CAR-T-cell target is BCMA, a member of the tumor necrosis factor receptor superfamily (TNFRSF17) whose preferential expression in plasma cells rather than hematopoietic stem cells makes it an ideal antigen target.^40^ In March 2021 and February 2022, the FDA approved 2 anti-BCMA CAR-T-cell products for MM treatment: ide-cel/Abecma and cilta-cel. In June 2023, the National Medical Products Administration approved a third CAR-T-cell product for MM treatment, CT103A. A phase II study showed a 73% response rate among patients treated with ide-cel, a 33% CR rate, and a median PFS of 8.8 months.^41^ In the latest phase III trial, the median PFS was 13.3 and 4.4 months, and the response rate was 71% and 42% in the ide-cel and standard regimen groups, respectively.^42^ A phase Ib/II trial designed to evaluate the safety and clinical activity of cilta-cel showed an overall response rate of 97%, a CR rate of 67%, common adverse events, and a 95% incidence of CRS; there was only 9% of severe CRS and no disease progression in approximately 60% of patients at 2 years.^43,44^ The FUMANBA-1 trial of CT103A showed an ORR of 95% and partial response of 4%, with a favorable safety profile.^45^ Presented at the 2023 ASCO Annual Meeting, the updated data showed significant improvements in treatment efficacy. The overall response rate surged to 96.0%, accompanied by a notable 74.3% rate of stringent CR (sCR). Impressively, 9 of 12 subjects with prior CAR-T-cell therapy achieved remission, with 5 achieving an sCR, 4 of whom sustained sCR for over 18 months.^46^ Further research is required to validate these findings.

In addition to BCMA, alternative targets have been identified including CD138, CD38, CD19, GPRC5D, SLAMF7(CS1), APRIL, TACI, CD229, CD56, MUC1, NKG2D ligand, integrin b7, kappa light chain, FcRH5, CCR10, and CD44v6.^47^ POLARIS was the first human, single-center, single-arm, first-arm trial of GPRC5D-targeted CAR-T cells (OriCAR-017) exclusively to enroll Chinese patients, resulting in an overall response rate of 100% and a CR rate of 60%, with no serious adverse reactions.^48^ O’Neal et al developed CAR-T cells targeting CS1 (SLAMF7), which showed efficacy in a high-tumor-load myeloma model, despite the fact that CD8^+^ CS1-expressing CAR-T cells killed CD8^+^ CS1 cells.^49^ A phase I trial of humanized bispecific BM38 CAR-T cells with BCMA and CD38 showed a response rate of 87%, a CR rate of 52%, and a median PFS of 17.2 months.^50^ Current CAR-T-cell therapy is typically used late in MM treatment; the preparation process is slow and costly, and relapse is inevitable in some patients. Exploring new targets to reduce CAR-T-cell toxicity and improve their safety is a future direction for development.

#### 2.4.4 Mantle cell lymphoma

With the exception of some patients undergoing allogeneic bone marrow (BM) transplantation, the curability of mantle cell lymphoma (MCL) is uncertain. The prognosis is poor after standard chemotherapy, with a median survival of approximately 3 years, and long-term disease-free survival is rare.^51,52^ CAR-T-cell therapy is a new and valuable treatment modality. The currently marketed CAR-T-cell product targeting R/R MCL is brexucabtagene autoleucel. ZUMA-2, a phase II multicenter clinical study, showed an objective response rate of 91% in all 68 treated patients after a median follow-up of 35.6 months, with a complete response of 68%.^53^ The TRANSCEND study reported an ORR of 84% and a CR of 59% in patients with R/R MCL treated with liso-cel.^54^

### 2.5 Adverse effects and toxicity

#### 2.5.1 Cytokine release syndrome

CRS, the most common adverse reaction to CAR-T-cell therapy, is an inflammatory state triggered by the release of inflammatory cytokines and chemokines from CAR-T cells upon binding to the corresponding target antigens.^55^ CRS develops in 55.3% of patients with hematologic malignancies after CAR-T-cell treatment.^56^ The American Society for Transplantation and Cellular Therapy (ASTCT) defines CRS as “a supraphysiological response following any immunotherapy that results in the activation or engagement of endogenous or infused T-cells and/or other immune effector cells.” The pathogenesis of CRS is currently unclear. IL-6 and IL-1, derived from monocytes, macrophages, and dendritic cells, play key roles.^57^ A study found that CAR-T cells activate gasdermin E (GSDME) to mediate cellular scorching and, subsequently, CRS by releasing large amounts of perforin and granzyme B.^58^ Animal experiments have shown that knocking down GSDME, destroying macrophages, or blocking the activation of GSDMD prevents the development of CRS.^59^

The symptoms may be progressive, the first of which is fever, which can occur hours or days after cell infusion,^60^ as well as hypotension, capillary leakage (hypoxia), and end-organ dysfunction.^57^ Other symptoms resemble those of influenza and include myalgia, headache, stiffness, malaise, and anorexia. Low-grade CRS is self-limiting, whereas other patients may present with hypotension, sepsis, and capillary-leak syndrome, which are further exacerbated by shock, disseminated intravascular coagulation (DIC), and multi-organ failure.^61,62^ Its progression and severity are generally considered to be related to factors such as disease burden, CAR structure and dose, and lymphatic-depletion conditioning regimens.^63^ The duration of CRS varies, depending on the intervention, and usually resolves completely 2 to 3 weeks after CAR-T-cell infusion.^62^

The most important step in the management of CRS is to use CAR-T-cell therapy only in patients who can tolerate CRS and provide appropriate supportive care and interventions, including direct targeting and non-specific immunosuppressive strategies, based on the patient and the CAR-T-cell product.^63,64^ Tocilizumab, an IL-6 receptor antagonist, is the only FDA-approved therapy for CAR-T-cell–associated CRS.^65^ Some studies have shown that early treatment with tocilizumab and/or corticosteroids reduces the incidence of CRS of grade 3 or higher.^66^ Siltuximab is a potential alternative to tocilizumab with a lower risk of exacerbating neurotoxicity; however, additional clinical data validating this are lacking. Anakinra, an IL-1 receptor antagonist, has a reported capacity to limit the extent and duration of CRS and reduce additional doses of tocilizumab or steroids.^67^ Non-specific immunosuppressants, such as glucocorticoids, may alleviate CRS symptoms but may theoretically limit the expansion and persistence of CAR-T cells, thereby compromising their efficacy. Further clinical data are required to provide support for its efficacy and safety. Glucocorticoids remain the cornerstone of CRS treatment.^64^

Further research is needed to clarify the mechanisms of CRS, aiding its prediction and accurate identification, and developing reasonable management strategies. As CAR-T-cell therapy gains further prominence, increased clinical experience will enable clinicians to manage CRS according to standardized diagnostic and treatment strategies, thereby enhancing CAR-T-cell safety.

#### 2.5.2 Immune effector cell–associated neurotoxicity syndrome

ICANS is another common adverse reaction to CAR-T-cell therapy that can occur simultaneously with, after, or independently of CRS.^55^ Neurotoxicity develops in 37.2% of patients with hematologic malignancies following CAR-T-cell therapy.^56^ ASTCT defines ICANS as “a disease characterized by a pathological process involving the central nervous system that results in the activation or engagement of endogenous or infused T cells and/or other immune effector cells following any immunotherapy.”^57^ Early manifestations may include expressive aphasia and changes in writing, followed by progression to altered levels of consciousness, coma, seizures, weakness, and cerebral edema. The mechanism by which this occurs is unclear. Studies have shown that the expression of CD19 in brain wall cells is responsible for the neurotoxicity that occurs with CAR-T-cell therapy, suggesting a mechanism for targeting neurotoxicity in CD19-directed therapies.^68^

The treatment options for ICANS include fasting, maintaining hydration, nutritional support therapy, and improved neurological examinations.^61^ Glucocorticoids are considered the first-line treatment of choice for rapid relief of ICANS.^69^ Steroid-refractory ICANS can be treated with anakinra. This resulted in a statistically significant and rapid reduction in fever, inflammatory cytokines, and biomarkers associated with ICANS/CRS.^70^ Levetiracetam prophylaxis may be used in patients with a history of seizures or central nervous system (CNS) diseases. Benzodiazepines and additional anti-epileptic drugs (levetiracetam and phenobarbital) are recommended for the treatment of active seizures.^55^

ICANS is a common complication of CAR-T-cell therapy with progressive symptoms, and early diagnosis and prevention are important tools for its management. Continuous improvements in measures to control adverse reactions can further highlight the advantages of CAR-T-cell therapy.

#### 2.5.3 Other related toxicity

Severe on-target, off-tumor (OTOT) toxicity often occurs in clinical trials of CAR-T-cell therapy for solid tumors, the primary mechanism of which is CAR-T-cell–mediated recognition and cleavage of normal cells expressing targeted antigens. Although the current primary targets of CAR-T cells for solid tumors are tumor-associated antigens (TAAs), these are also expressed in non-malignant tissues. Current strategies to mitigate OTOT include: (1) modifying CAR components to improve the specific recognition of tumor cells by CAR-T cells; (2) reducing the toxicity of CAR-T cells through logic-gated switches to improve the specificity of cell death; (3) use of drugs to limit CAR-T-cell function by regulating their signaling pathways or the CAR expression level.^71^

Owing to the targeted non-tumor effects of anti-CD19 CAR-T cells on normal cells, B-cell dysplasia often occurs, leading to hypogammaglobulinemia and subsequent manifestations of frequent infections. B-cell dysplasia has been reported for up to 5 years after anti-CD19 CAR-T-cell treatment for B-ALL.^72^ Infections are very common after CAR-T-cell therapy, mostly occurring within 1 to 2 years, and the incidence of infection is approximately 55%. The mortality rate of co-infections during the CRS response period is high; therefore, infection prevention and control are the highest priorities for CAR-T-cell therapy.^73^ Hemocytopenia includes anemia, thrombocytopenia, leukopenia, and neutropenia. In addition, CAR-T-cell therapy causes adverse reactions, including DIC, phagocytic syndrome, tumor-lysis syndrome, and graft versus host disease (GVHD).

### 2.6 Relapse

Relapse after CAR-T-cell therapy for B-cell malignancies is defined as the persistence of tumor cells after achieving CR via T-cell infusion. It occurs in approximately 40% to 60% of patients; in R/R ALL, 30% to 60% of patients relapse after CAR-T-cell therapy, of which 10% to 20% are CD19-negative relapses.^74,75^

Failure of CAR-T-cell therapy is most often attributed to antigen escape, that is, antigen-negative relapse; however, antigen-positive relapse also occurs frequently, which may be associated with CAR-T-cell intrinsic factors.^76^ Thus, the tumor cells themselves, tumor microenvironment (TME) and CAR-T cells may influence relapse. The inherent heterogeneity of tumor cells lays the foundation for the clonal evolution and malignant progression of tumors. Prior to CAR-T-cell infusion, tumor cells with low or negative antigen expression levels may escape and undergo secondary clonal expansion. The impaired function and diminished persistence of CAR-T cells in vivo following infusion may also lead to relapse.^74^ Point mutations in CD19 exon 3 can affect the epitopes recognized by CD19 FMC63 CAR-T cells and induce CD19-positive relapse in patients with high-grade B-cell lymphoma.^77^ Most of the single-stranded variable fragments (scFv) in CAR are derived from mice and have high antigenicity, low persistence, and a high recurrence rate.^78^

## 3. WHERE ARE WE HEADING?

### 3.1 Application expansion

CAR-T-cell therapies for hematologic malignancies, including HL, T-cell lymphoma, and acute myeloid leukemia (AML), remain in the exploratory phase of investigation. Although the scope of CAR-T-cell therapy for solid tumors encompasses nearly all tumor types, its clinical implementation remains constrained by the challenges posed by tumor antigen diversity and the heterogeneous nature of the TME. Therefore, the translation of CAR-T-cell therapies into clinical practice for these indications is yet to be realized. Furthermore, the applications of CAR-T cells have expanded to include non-neoplastic conditions such as autoimmune diseases, infections such as HIV, and myocardial fibrosis.

#### 3.1.1 Hodgkin lymphoma

HL is a B-cell lymphoma characterized by a small number of malignant cells and a large number of immune effector cells in the TME. The incidence of HL is low, with a rate of approximately 2.7 to 2.8 cases per 100,000 people in the United Kingdom and the United States, with a higher incidence in men than in women, and the highest incidence in the young and in individuals over 60 years of age.^79^ Most patients with classical HL are cured with first-line therapy; however, approximately 15% develop primary refractory disease or relapse after an initial treatment response.^80^ The SOC for patients in whom first-line therapy fails is high-dose chemotherapy followed by autologous HSCT; approximately 50% of patients relapse after transplantation, with a poor prognosis.^81^ Anti-CD30 CAR-T cells have entered clinical trials for the treatment of HL, and no toxicities were observed in any of the 7 patients with relapsed HL in a phase I study. One patient developed a complete response (after a second infusion) lasting >2 years.^82^ Ramos et al conducted 2 parallel phase I/II clinical trials with an overall ORR of 62%, no dose-limiting toxicity, mild hematologic toxicity, and a CRS incidence of 24%.^83^

#### 3.1.2 T-cell malignancies

T-cell malignancies are a broad spectrum of heterogeneous neoplasms, including precursor T-cell malignancies, such as T-lymphoblastic leukemia/lymphoma (T-ALL/LBL), and mature T-cell malignancies, such as T-cell large granular lymphocytic leukemia, adult T-cell leukemia/lymphoma, T-cell prolymphocytic leukemia, and numerous peripheral T-cell lymphomas (PTCLs).^84^ T-ALL is a malignancy of the lymphatic system that accounts for 10% to 15% of ALL cases in children and approximately 25% of cases in adults.^85^ T-lymphocytic lymphoma (T-cell lymphoma) is a type of NHL with biological characteristics similar to those of T-ALL, which is classified as cutaneous T-cell lymphoma or PTCL. In T-cell lymphoma, prognosis is poor, usually because of drug resistance and patient intolerance of chemotherapy regimens.^86^

Unlike the situation in B-cell malignancies, CAR-T-cell therapies face challenges in fighting malignant T-cell self-mutilation, T-cell regeneration, and product contamination.^87^ There are numerous CAR-T-cell targets for T-cell malignancies, including CD7, CD7, CD99, and Vβ8, all of which are currently in the research stage. CD7 is a transmembrane glycoprotein of the immunoglobulin superfamily that is expressed in a high proportion of T-ALL and T-cell lymphomas.^88^ Lu et al reported an open, single-arm phase I clinical trial of naturally selected CD7 CAR-T (NS7CAR). Of 20 patients with R/R T-ALL and T-cell lymphoma, 19 achieved minimal residual disease-negative CR in the BM on day 28, and 14 received allogeneic HSCT after NS7CAR infusion and have not relapsed to date. Grade 3 CRS occurred in only 1 patient.^89^ In September 2022, Hu et al reported on a phase I clinical trial using CD7 general-purpose CAR-T cells for the treatment of T-cell malignancies, with an overall response rate of 81.8% and a complete response rate of 63.6% in 11 evaluable patients with no dose-limiting toxicity, GVHD, ICANS, or severe CRS (≥grade 3).^90^ Shi et al reported the use of anti-CD99 CAR-T cells in T-ALL and showed effective inhibition of proliferation and direction of apoptosis in tumor cells, in vitro and in vivo, while exhibiting no cytotoxicity toward normal cells.^91^ Li et al reported that anti-Vβ8 CAR-T cells were able to identify and kill all Vβ8^+^ malignant T-cells generated by clonal expansion while retaining malignant or healthy Vβ8-T cells, resulting in sufficient T-cell–mediated cellular immunity.^92^

#### 3.1.3 Acute myeloid leukemia

AML is a heterogeneous hematologic malignancy and the most common form of acute leukemia in adults. It is characterized by the clonal expansion of myeloblasts in the peripheral blood, BM, and/or other tissues.^93^ In the United States, the annual incidence of AML is 4.3 per 100,000 individuals, with a slightly higher prevalence in males than in females. AML accounts for up to 62% of leukemia deaths among all subtypes.^94^ Following conventional chemotherapy, the recurrence rate is 10% to 60% in young adults and 40% to 60% in the older population (>60 years), with a 5-year patient survival rate of approximately 27.4%.^95^

Jetani et al developed a CAR-T cell targeting Siglec-6, which preclinical model studies showed were anti-leukemic-responsive and could induce complete tumor remission in xenograft AML models.^96^ Hebbar et al reported a GRP78-CAR-T cell with significant anti-AML activity in vivo without toxicity to hematopoietic progenitor cells.^97^ A phase I study of CLL-1 CAR-T cells showed complete response (CR)/CR with incomplete hematologic recovery (CRi) rate of 70% and an OS rate of 60%. All patients experienced CRS without neurological toxicity.^98^ Sugita et al evaluated the potential of allogeneic gene editing of CAR-T cells to CD123 (UCART123) for the treatment of AML. UCART123 was effective in eliminating AML both *i*n vivo and in vitro without significant effects on normal cells.^99^ A phase I/II clinical trial on autologous anti–CLL-1 CAR-T cells showed that of 8 children with R/R AML, 4 achieved morphologic leukemia-free status without minimal residual disease, and 1 achieved CR.^100^ Wu et al reported a preclinical study on anti-CD70 CAR-T cells in AML, demonstrating potent cytotoxicity when cultured with CD70^+^ AML cell lines and potent anti-leukemic activity in a mouse model. However, this did not completely eliminate leukemia *i*n vivo.^101^ Currently, no ideal CAR-T-cell target for AML has been identified. Recent research has proposed the use of single-cell transcriptome profiling to predict target antigens expressed on malignant but not on healthy cells, including T-cells, to develop new CAR-T cells for AML treatment. Using publicly available RNA sequencing data profiles, including more than 500,000 single cells from 15 AML patients and tissues from 9 healthy patients, investigators computationally identified 2 potential new targets, CSF1R and CD86. CSF1R-CAR-T and CD86-CAR-T cells demonstrated robust efficacy in both AML cell lines and human-derived AML models in a functional validation trial.^102^

#### 3.1.4 Solid tumors

The indications for CAR-T-cell treatment of solid tumors are diverse and cover almost every tumor type. The key challenges can be summarized in 3 main components: recognition, trafficking, and survival. Solid tumor antigenic heterogeneity hinders the recognition of tumor cell–specific antigens. In contrast, the immunosuppressive TME interferes with T-cell activity in terms of both differentiation and exhaustion.^103,104^

A previous study showed that anti-GPC3-CAR-T cells effectively inhibited and eliminated tumors in a primary human hepatocyte xenograft model.^105^ Cao et al developed a novel bispecific GPC3-BiTE CAR that exhibited better anti-tumor activity than single-target CAR-T cells in an in vitro functional analysis and overcame antigen heterogeneity-induced tumor escape.^106^ Moghimi et al developed a GD2/B7H3 CAR-T cell model which demonstrated a high degree of specificity in inhibiting neuroblastoma growth and increased durability in vivo.^107^ A phase I mid-stage trial of CLDN18.2-targeted CAR-T cells (CT041) in gastrointestinal cancers showed an overall response rate and disease control rate of 48.6% and 73.0%, respectively. All the patients experienced ≥grade 3 hematologic toxicity, and no CRS ≥grade 3 or neurotoxicity was observed.^108^ Meister et al developed a multifunctional mRNA-based CAR-T cell that exhibited strong anti-glioma activity and showed no signs of toxicity in in vivo or in vitro assays.^109^

Li et al developed a synthetic zinc finger transcriptional regulator, synZiFTR, to regulate the *i*n vivo efficacy of CAR-T cells in a xenograft hematologic tumor model by programming the drug-dependent, post-delivery control of in vivo T-cell anti-tumor activity. Tamoxifen-inducible synZiFTR was used to regulate the expression of cytokines including IL-2 and IL-12 in vitro in a dose- and time-dependent manner, which safely and controllably enhanced CAR-T efficacy.^110^ Subsequently, Allen et al designed an enhanced Notch receptor-containing CAR-T cell and showed that the engineered synNotch-induced IL-2 circuit produced IL-2 locally, driving effective infiltration of CAR or TCR T-cells into immune-rejection tumor models of pancreatic cancer and melanoma, enhancing immune cell infiltration and reducing systemic toxicity.^111^ A preclinical study found that the local injection of CAR-T cells targeting mesothelin cleared residual tumor tissue in mice without significant toxic side effects.^112^

#### 3.1.5 Myocardial fibrosis

Cardiovascular disease constitutes approximately 31% of all deaths worldwide, and nearly all heart diseases associated with heart failure involve myocardial fibrosis.^113^ Fibrosis, characterized by excessive extracellular matrix protein deposition, is a fundamental pathological response to chronic inflammation with important pathophysiological implications in cardiovascular disease development.^114^ Therapeutic strategies for myocardial fibrosis are still in the research phase, and CAR-T-cell therapies have made significant progress. Rurik et al developed a method generating transiently engineered CAR-T cells in vivo, which reversed fibrosis and significantly improved heart failure in vitro.^115^ This offers potential not just for myocardial fibrosis but also for other fibrotic diseases. However, further studies are required to verify safety and efficacy.

#### 3.1.6 Autoimmune diseases

Autoimmune diseases arise from the immune system attacking the body’s own tissues and are usually classified as either organ-specific or systemic. Current conventional therapies include non-steroidal anti-inflammatory drugs, glucocorticoids, and disease-modifying anti-rheumatic drugs; however, complete elimination of the disease has proved elusive.^116,117^

CAR-modified T-cells can kill abnormal immune cells such as B-cells or antibody-secreting plasma cells in autoimmune diseases, offering a potential cure. Mougiakakos et al reported successful treatment using autologous CD19 CAR-T cells in a patient with severe refractory systemic lupus erythematosus (SLE) who presented with active lupus nephritis, achieving serologic and clinical remission and a decrease in the SLE disease activity index (DAI) score from 16 at baseline to 0 at follow-up, with no adverse events.^118^ Subsequently, Mackensen et al reported on 5 patients with refractory SLE treated with CAR-T-cells. All patients achieved SLE remission at 3 months, with a median SLE DAI score of 0 and achieved drug-free remission, with no relapses observed at follow-up; they also tolerated CAR-T-cell therapy well, with no or only mild adverse effects. These data suggest that CD19^+^ CAR-T-cell transplantation is feasible, tolerable, and efficient for treating SLE.^119^ Müller et al reported a second autoimmune disease cured with CAR-T anti-synthetase antibody syndrome. After 6 months of CAR-T-cell therapy, magnetic resonance imaging showed complete regression of the myositis lesions, disappearance of pneumonia, and drug-free remission.^120^

Currently, comprehensive long-term data on the effectiveness of CAR-T-cell therapy in the context of autoimmune disorders are lacking. Clinical applications primarily remain in the investigative stage, with limited cases undergoing clinical trials, thereby necessitating the continued evaluation of safety and long-term outcomes. Nonetheless, these promising results highlight the potential of CAR-T-cell therapy as a curative measure for autoimmune diseases.

#### 3.1.7 Infectious diseases

Among infectious diseases, AIDS is the main focus of investigation for CAR-T-cell therapy. There is no cure for this disease, and patients remain on lifelong medication. Although the advent of antiretroviral therapy has greatly reduced the morbidity and mortality associated with HIV infection, a stable reservoir of HIV in latently infected cells requires lifelong adherence to treatment, and discontinuation of antiretroviral therapy can lead to rapid reactivation of the virus.

Liu et al developed a broadly neutralizing antibody-derived CAR-T-cell therapy that produces specific cytotoxic activity against HIV-1–infected cells and effectively removes HIV-reactivated infected CD4^+^ T lymphocytes.^121^ Recent studies have shown that peripheral intravenous injection of anti-HIV duoCAR-T cells can localize to sites of active HIV infection in the spleens of humanized mice and eliminate primary HIV-infected cells.^122^

### 3.2 New targets

Currently, CAR-T-cell therapy is still in the research stage for AML, HL, T-cell malignancies, and solid tumors, in addition to its success in B-cell malignancies. It is hoped that the development of new targets will help advance CAR-T-cell therapy in the treatment of these diseases. For example, CD5^+^ CAR has been effective in preclinical studies targeting a variety of T-cell cancers. Feng et al developed CD5-IL15/IL15sushi CAR-T cells that were sufficient to ablate CNS lymphocytes within a few weeks, and the patients experienced only transient T-cell dysplasia events. These results suggest that this may be a safe and effective treatment for T-cell malignancies, especially those with CNS involvement.^123^ epidermal growth factor receptor (EGFR) is highly expressed in triple-negative breast cancer (TNBC) and may be a relevant immunotherapeutic target; anti-EGFR CAR-T-cells showed potent and specific anti-tumor activity against TNBC in vivo and in vitro.^124^ Another study showed that second-generation anti-EGFR CAR-T cells were effective in eradicating TNBC brain metastases in mice and improving their survival rates,^125^ further highlighting EGFR as a promising target for TNBC treatment.

**Figure 2.**
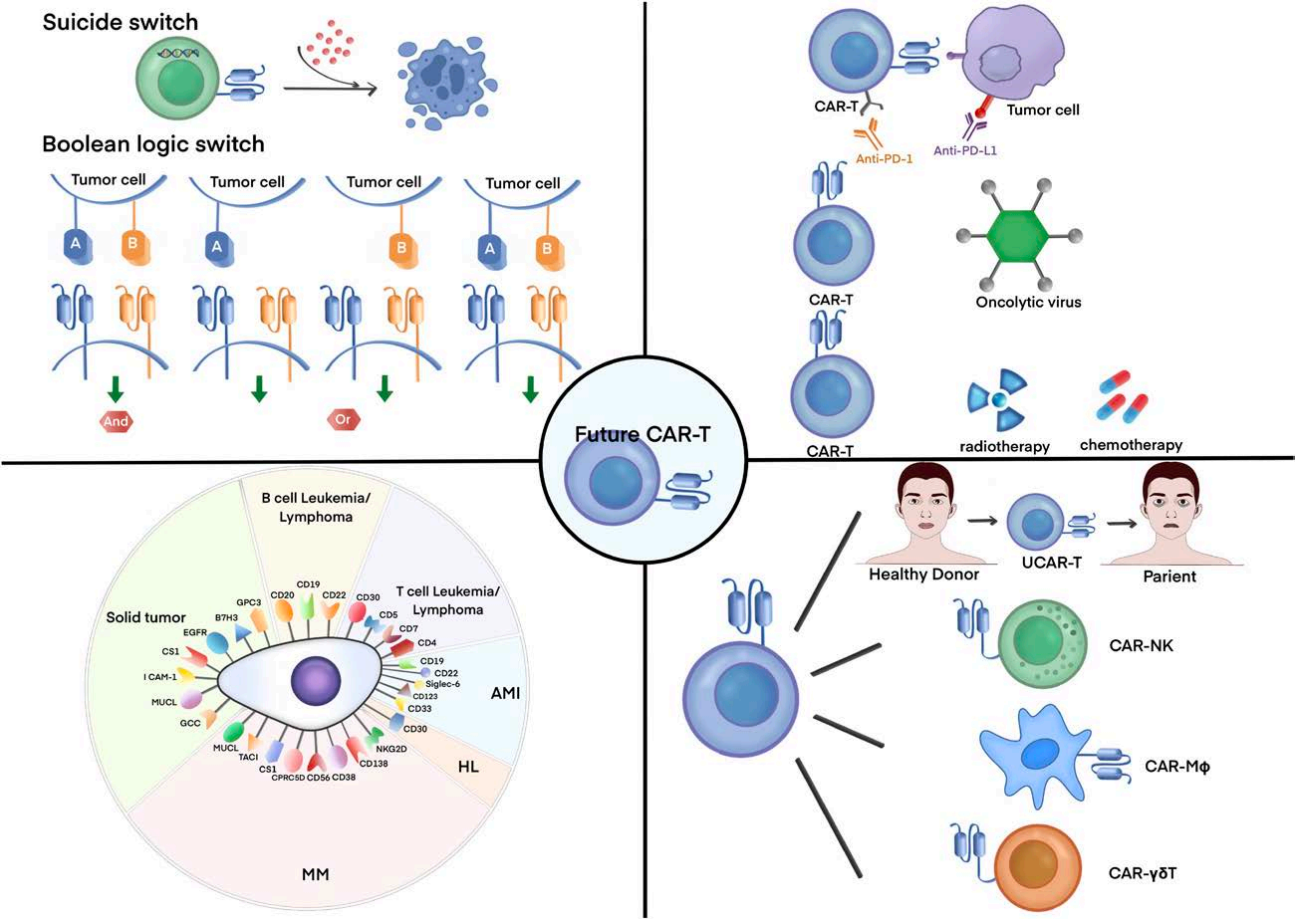
Where are we heading in the field of CAR-T? (A) New targets for CAR-T-cell therapy for B-cell leukemia/lymphoma, T-cell leukemia/lymphoma, AML, HL, MM, and solid tumors; (B) New design for CAR-T cell, utilizing suicide or logic-gated switches to improve their safety and controllability; (C) CAR-T cells in combination with immune checkpoint inhibitors, lysoviruses, or radiotherapy; (D) Next-generation CAR-Ts, including UCAR-T, CAR-NK, CAR-MΦ, and CAR-γδ T. AML = acute myeloid leukemia, CAR = chimeric antigen receptor, CAR-MΦ = CAR macrophages, HL = Hodgkin lymphoma, MM = multiple myeloma, NK = natural killer cell, UCAR-T = universal CAR-T.

Single-target CAR-T cells typically encounter problems such as antigen escape and clonogenic proliferation-negative recurrence.^126^ Targeting two or more tumor-associated molecules may not only prevent the immune escape of tumor cells but may also overcome antigenic heterogeneity in solid tumors. Bivalent CD19/CD22-4-1BB CAR-T cells targeting CD19 and CD22 showed a CR rate of 60% and a BM CR of 80% in 20 patients with R/R B-ALL in a phase I dose-escalation trial. The novel optimized CD19.28ζ/CD22.BBζ double cis-trans CAR-T-cells containing EF1α promoter cells enhanced the ability to target CD22 while improving amplification persistence, preventing antigen escape, and maintaining remission.^127^ Bispecific CAR-T cells targeting BCMA and CD38 have shown a response rate of 87.5% and a sCR rate of 81.3% in clinical trials, with low recurrence rates and controlled CRS.^128^ Tian et al developed a BiCisCAR-T cell line targeting both GPC2 and CD276, which was able to overcome neuro antigen heterogeneity in blastomas by targeting multiple TAAs and antigenic heterogeneity in solid tumors.^129^ Dai et al showed that CD5/CD7 bispecific CAR-T cells exhibited good tumor-killing ability in vitro and good proliferation under continuous antigen stimulation. Additionally, tandem dual-targeted CARs were more effective than parallel dual-targeted CARs in activity and preventing tumor escape (Fig. 2).^130^

### 3.3 New CAR designs

Suicide switching can eliminate CAR-T cells from the body and prevent potentially life-threatening adverse events. Genome editing technology is an important driver of therapeutic advances in CAR-T cells.^131^ Inducible caspase 9 (IC9) is a suicide gene that can be activated by an inert, small biomolecule, AP1903. Inclusion of IC9 in CAR construct designs can serve as an effective safety switch to control malignant CAR^+^ B-cells.^132^

Boolean logic gating switches are the mathematical operators “IF/THEN,” “AND,” “OR,” and “NOT.” The use of logical gating switches in CAR-T-cell design can further control their activation, improve the specificity of cell killing, and reduce toxic side effects.^71^ Logically gated CAR-T cells targeting carcinoembryonic antigen and mesothelin showed specific toxicity against tumor cells expressing both antigens, without affecting the growth of single antigen-positive tumor cells.^133^

Traditional CAR-T-cell manufacturing takes 2 to 3 weeks, offering opportunities for rapid leukemia-cell growth, thus affecting efficacy and safety. There are still impediments to the pace of development of real-world CAR-T-cell accessibility, and autologous CAR-T cell preparation time remains a significant limiting factor for patients receiving treatment.^134^ Zhang et al established the FasTCAR autologous cell CAR-T technology platform that allows the production of CAR-T cells (GC007F) within 24 hours and is available for clinical application 8 days after blood collection. In a multicenter phase I interventional clinical study, all 18 patients who were evaluated for efficacy achieved CR on day 28, with a CRS incidence of 95.2%. Compared with C-CAR-T cells, this therapy is safer and more controllable, with greater in vivo expansion capacity, longer retention time, and better efficacy.^135^

### 3.4 Next-generation CARs

#### 3.4.1 Universal CAR-T

Autologous CAR-T-cell production is costly and has a long production cycle, and patient lymphocytes are few and poorly functional; UCAR-T cells from healthy donors appear to overcome these limitations, but can induce GVHD and their persistence is disrupted by the host immune system.^25^ Multiplex genomic CRISPR/Cas9-edited UCAR-T cells exhibit potent anti-tumor activity in vitro and in animal models, similar to non-gene-edited CAR-T cells.^136^ CRISPR/Cas9-designed universal CD19/CD22 CAR-T cells exhibit a controlled safety profile and outstanding hemocytosis-free activity. Generic dual-target CAR-T-cell therapies may provide alternative treatments for patients with R/R ALL.^38^ Those produced by TALEN-mediated gene editing can avoid natural killer cell (NK) and alloreactive T-cell attacks and extend their persistence and anti-tumor activity at the level of cytotoxicity in vivo and in vitro in NK cells, respectively.^137^

#### 3.4.2 Other substitutable cells

Owing to the following factors—their natural killing function; abundant sources such as the NK92 cell line, umbilical cord blood, peripheral blood, and induced pluripotent stem cells; and the absence of GVHD induction—CAR-NK cells are currently being explored.^25^ Liu et al used anti-CD19 CAR-NK cells in 11 patients with recurrent or refractory CD19-positive cancer and showed a remission rate of 73% and a CR rate of 64%.^138^ For patients unsuitable for single-cell isolation, primary human hematopoietic stem and progenitor cells were used to generate functional CAR macrophages (CAR-MΦs) in vitro, with the same targeted activation as CAR-T-cells.^139^ Chen et al subsequently used local injection of CAR-MΦs targeting glioma stem cells to effectively eliminate tumor cells and suppress post-operative glioblastoma recurrence.^140^ γδ T-cells lack homozygosity, can be safely used for haplo-identical transplantation, and mediate natural anti-tumor responses. Rozenbaum et al found that the CAR transduction efficiency of γδ T-cells was as high as that of standard CAR-T cells during production; in terms of anti-tumor effects, CD19-directed γδ T-cells were effective against both CD19-positive and negative leukemic cells.^141^

### 3.5 Changing the administration plans of CAR-T-cells

#### 3.5.1 Optimizing drug delivery

Increasing the duration of action of a single infusion or multiple infusions of CAR-T cells is an effective way to improve their persistence.^142^ Li et al developed a CAR with recyclable capacity (CAR^KR^-T) and showed significant inhibition of CAR downregulation and enhancement of internalized CAR recycling to the cell surface, promoting long-term killing ability.^143^ CD19 CAR-T-cell therapy is highly effective in R/R B-cell malignancies but mostly fails to induce durable responses. A second infusion (CART2) could possibly improve outcomes. Higher overall response rates, longer PFS, and a lower incidence of serious toxicity were observed with the second infusion than with the first.^144^ A second infusion of CAR-T cells in patients with R/R B-ALL was beneficial in some patients, whereas in others, reduced CAR-T-cell expansion and antigen downregulation hindered a robust response to CART2.

Intravenous injection is the primary mode of CAR-T-cell administration; however, this is not ideal for treating solid tumors. Intrathecal injection of CAR-T cells can treat recurrent medulloblastoma and ventricular meningioma in a local area, bypassing the blood-brain barrier and showing superior anti-tumor effects in mouse models.^145^ Grosskopf et al fabricated an injectable hydrogel that controlled the co-delivery of CAR-T cells and stimulatory cytokines, which effectively eliminated tumors with a 100% response rate; it did not induce adverse inflammatory responses, and was completely degraded in vivo within weeks.^146^

#### 3.5.2 Combination therapies

CAR-T combination therapy combines CAR-T-cell therapy with other options, such as radiotherapy, chemotherapy, immune checkpoint inhibitors, and oncolytic viruses, which can improve its safety and efficacy.

Radiotherapy and chemotherapy are the traditional tumor-treatment modalities. Zhou et al found that combining CAR-T cells targeting EGFR with radiation therapy effectively enhanced the immune function and anti-tumor efficacy in immunodeficient in situ TNBC mice.^147^ Effective chemotherapy reduction improved the short-term ORR and long-term OS of CAR-T-cell therapy in R/R DLBCL patients with high-tumor loads, resulting in outcomes comparable with those of patients with low tumor loads.^148^

PD-1 suppression of T-cell function in the TME restores the effector function of CD28 CAR-T cells via PD-1 antibody checkpoint blockade, cell-intrinsic PD-1 shRNA blockade, or PD-1 dominant-negative receptors.^149^ Simultaneous downregulation of 3 inhibitory immune checkpoint receptors—PD-1, Tim-3, and Lag-3—in CAR-T cells enhanced tumor infiltration and inhibition of tumor growth.^150^ Decitabine-treated CAR-T cells exhibited enhanced anti-tumor activity and longer-lasting anti-tumor potential both in vivo and in vitro.^151^ Bruton tyrosine kinase (BTK) is a key mediator of B-cell receptor–dependent cell growth and is upregulated in a variety of B-cell malignancies. BTK inhibitors (BTKis) are highly effective in the treatment of MCL and chronic lymphocytic leukemia. Ibrutinib, the first BKTi product on the market, enhances efficacy in combination with anti-CD19 CAR-T cells.^152,153^ The concomitant administration of CAR-T-cell therapy and ibrutinib led to a higher ORR (83%) than CAR-T-cell therapy alone (56%).^154^ In mouse experiments, ibrutinib had a synergistic effect when combined with CD19 CAR-T cells; the CAR-T cells in the mice were significantly expanded, and the proportion of expansion was higher than with CAR-T-cell treatment alone, significantly reducing the tumor load.^153^

Oncolytic virotherapy is a new form of cancer treatment that uses lysing viruses to directly attack or express viral antigens in infected cancer cells, leading to recognition and destruction by cytolytic T-cells. Evgin et al loaded CAR-T cells with vesicular stomatitis or eutherian virus for treating solid tumors in mouse models. Systemic delivery greatly enhanced the efficacy of CAR-T cells in treating melanoma and glioma tumors and improved survival.^155^

## 4. CONCLUSIONS

In summary, the success of CAR-T-cell therapy in hematologic malignancies is well documented, and its potential in addressing other disease areas is evident. However, the challenges related to treatment-related toxic reactions and relapses require further investigation. Strategies aimed at identifying novel targets, refining or designing new CAR structures, altering the administration approach, and exploring combination therapies have the potential to enhance the safety and efficacy of CAR-T cells while mitigating toxicity and reducing relapse. Furthermore, investigations into alternative immune cell therapies, such as CAR-NK cells, are underway. The future holds promise for the development of CAR-cell therapies that can be tailored for precise and personalized treatment of a range of diseases.

## ACKNOWLEDGMENTS

The authors gratefully acknowledge the financial support provided by grants from the National Natural Science Foundation of China (No. 82170181), Beijing Natural Science Foundation (No. 7222027), and the National Key R&D Program of China (Grant No. 2022YFF1502000) to Liang Wang.

## AUTHOR CONTRIBUTIONS

J.Y.W. and L.W. made a substantial contribution to the conception of the work, drafted the work, revised it critically, and approved it for publication. Both authors contributed to the article and approved the submitted version.
